# Antibody-Mediated Nodo- and Paranodopathies

**DOI:** 10.3390/jcm13195721

**Published:** 2024-09-25

**Authors:** Valérie Quinot, Kevin Rostasy, Romana Höftberger

**Affiliations:** 1 Division of Neuropathology and Neurochemistry, Department of Neurology, Medical University of Vienna, 1090 Vienna, Austria; valerie.quinot@meduniwien.ac.at; 2 Department of Pediatric Neurology, Children’s Hospital Datteln, University Witten/Herdecke, 45711 Datteln, Germany; kevin.rostasy@uni-wh.de

**Keywords:** paranodopathy, nodopathy, neurofascin 155, neurofascin 186/140, pan-neurofascin contactin 1, Caspr1, CNTN1/Caspr1

## Abstract

The recent discovery of pathogenic antibodies targeting cell adhesion molecules of the node of Ranvier has prompted efforts to develop a new classification for a subset of antibody-mediated peripheral neuropathies. These autoimmune nodo- and paranodopathies encompass epitopes such as neurofascin 155, neurofascin 186, contactin-1, and contactin-associated protein 1, with a high likelihood of involving additional yet unidentified proteins. So far, the investigation of this subset of patients was primarily focused on adults, with only rare reports of pediatric cases. Low awareness among pediatricians and insufficient availability of appropriate diagnostic methods in many laboratories may mask a higher pediatric incidence than currently observed. Diagnosis is made by transfected cell-based assays and ELISA to characterize the specific target antigen and antibody subclass that provides insight into the pathophysiology. Clinical features often resemble those of CIDP or GBS in adults, whilst in pediatric patients, although rare, an atypical CIDP phenotype has predominantly been reported. Yet, in contrast to classical immune-mediated neuropathies, the clinical course is usually rapidly progressive, and response to classical first-line therapy often poor. Although electrophysiological signs of demyelination are observed, segmental demyelination and inflammation are not present on pathological examination. Rather, few neuropathological reports demonstrate features of axonal neuropathy without signs of true de- or remyelination. This review aims to summarize recent findings on such nodo- and paranodoneuropathies, shining light on features of these disorders in pediatric patients, a still little-explored field with only a few reports currently present.

## 1. Introduction

Peripheral neuropathies are traditionally classified into primarily axonal and demyelinating, with their pathophysiological characteristics delineated by electrophysiological and histopathological features [1]. In 2013, Uncini and colleagues advanced this dichotomy by introducing the term “nodo- and paranodopathy”, which has since been used to describe a group of neuropathies caused by antibodies targeting gangliosides [1]. Shortly thereafter, the concept was extended to include neuropathies in which the nodes represent the primarily involved site of pathology, with various defining etiologies including inflammatory, ischemic, toxic, metabolic, and immune-mediated factors [2]. Eventually, antibodies targeting peptide structures of the node and paranode including contactin1 (CNTN1), contactin-associated protein 1 (Caspr1), and different neurofascin (NF) isoforms (NF155, NF186/140) were identified in subsets of patients presenting clinical features reminiscent of Guillain–Barré syndrome (GBS) and chronic inflammatory demyelinating polyradiculoneuropathy (CIDP) [3,4,5]. It was, however, quickly observed that among other features, the clinical phenotype and therapy response differed from that of GBS and CIDP, ultimately leading the European Academy of Neurology and Peripheral Nerve Society (EAN/PNS) guidelines to introduce a new diagnostic category in 2021 and coining the term “autoimmune nodopathy” (AN) [6].

Highlighting the importance of this new categorization, the number of studies aimed at further characterizing clinical and pathophysiological aspects of AN in detail has increased exponentially, with a growing number of reviews emerging consequently [7,8,9,10]. Although certain features, including a tendency to gender-specific incidences, age-relatedness, and pathognomonic symptom constellations in specific ANs, have already been summarized in detail, other aspects remain underinvestigated. In this review, we provide an updated summary of clinical presentation, pathophysiology, diagnostics, and therapy, as well as their implications for clinical practice, with a focus on pediatric features of ANs in each section where possible, thereby drawing attention to a subgroup of patients who strongly benefit from increased awareness among physicians.

## 2. Anatomy of the Node of Ranvier and Its Antibody Epitopes

In the peripheral and central nervous system (PNS and CNS), myelinated nerves are arranged into uniquely defined regions, i.e., nodes of Ranvier, and adjacent paranodal and juxtaparanodal junctions, each characterized by different compositions of cell adhesion molecules (CAMs), signal transduction proteins, and cytoskeletal scaffolding components [11]. Densities of a variety of ion channels found in these domains vary respectively, thereby ensuring the propagation of action potentials via saltatory conduction. Nodes—the uninsulated myelin-sheath-free regions of the axolemma—comprise regions with a high density of voltage-gated sodium channels. Recruitment and stabilization of these ion channels are propagated by the 186-kDa isoform of neurofascin NF186 located on the axolemma, which, in the PNS, clusters with the Schwann cell microvilli-secreted protein gliomedin extracellularly, whilst interacting with nodal scaffolding proteins such as ankyrin G and βIV-spectrin in the axonal cytosol [11,12].

At the paranode, a further isoform of neurofascin, NF155, expressed on the terminal myelin loops of Schwann cells in the PNS and oligodendrocytes in the CNS, interacts with heterodimers of Caspr1 and CNTN1 [11]. Heterodimerisation occurs at the axonal endoplasmic reticulum, with subsequent complexes sent to the paranode directly, ultimately forming septate-like junctions that ensure myelin insulation ibid [13,14].

Similarly, at the juxtaparanode, complexes made up of contactin-associated protein 2 on the axolemma, and contactin 2 alias transient axonal glycoprotein 1 (TAG1), anchored on both glial and axonal membranes interact with high densities of voltage-gated potassium channels involved in repolarization of the membrane potential [15,16]. Moreover, gangliosides located in all three domains and on both glial and axonal membranes, as well as disintegrin and metalloproteinases (ADAMs) and a plethora of further molecules, including Nr-CAM, syndecan-3/4 and laminins, are likewise involved in structure and function of these domains [11]. 

Numerous studies have sought to identify specific nodal, paranodal, and juxtaparanodal proteins involved in neuropathies, recognizing the likelihood that additional, yet unidentified proteins contribute to these conditions. Indeed, various candidates have been identified, such as anti-gliomedin antibodies in multifocal motor neuropathy [17] and anti-moesin antibodies in cytomegalovirus-related Guillain–Barré Syndrome [18]. This review, however, focuses on the most common epitopes of the nodal and paranodal regions, i.e., NF155, NF186/140, CNTN1, and Caspr1.

## 3. Antibody Detection

The updated EAN/PNS CIDP diagnostic guidelines from 2021 recommend testing for nodal and paranodal antibodies in the serum of patients fulfilling the criteria of CIDP, initially presenting with acute or subacute aggressive onset, previously diagnosed as GBS or acute onset-CIDP, as well as in patients with a distinct set of symptoms including low-frequency tremor, ataxia, cerebellar symptoms, and predominant distal weakness [6]. Moreover, certain antibody-specific symptoms may already hint at the respective AN prior to antibody testing (see individual ANs). Respiratory failure, cranial nerve involvement, nephrotic syndrome, strongly elevated CSF protein levels and resistance to the standard intravenous immunoglobulin (IVIg), and corticosteroid therapy should also prompt antibody diagnostics [6].

Antibodies in question should be tested in serum via cell-based assays (CBAs) using mammalian expression vectors encoding human epitopes. However, some studies have shown false positive antibody status on fixed cells using tagged vectors with individuals harboring antibodies directed against specific tags, as was observed with myc-DDK-tagged NF155 plasmids in NF155 testing [19]. Confirmation of the results with the enzyme-linked immunosorbent assay (ELISA) and/or teased-nerve immunohistochemistry is also strongly recommended, and CBA or ELISA may also be used for isotype detection [6,19]. In addition, further binding experiments conducted on myelinating neuron cultures may be helpful in distinguishing distinct binding clusters [20]. Repetition of testing may be of use if patients are initially seronegative but involvement is clinically suspected and the sample is taken after treatment or in a remission phase [21,22]. While CSF parameters may be supportive of diagnosis, the value of antibody testing herein is yet to be determined [6,21,23]. 

However, not all laboratories have equivalent resources for antibody testing. Moreover, as the initial detection of these antibodies and the assumed clinical correlations were based on findings obtained using CBAs, in part combined with ELISA and teased nerve fibers, it is important to consider that results yielded using diagnostic methods other than those mentioned above may differ in comparability [21]. This issue is of particular relevance regarding studies using Western blots, as conformational epitopes may not be identified, and other irrelevant domains may instead exhibit reactivity. In addition, in-house methods may lead to inter-laboratory variability, and the range and composition of antigens tested may, in part, account for differences in profiles and frequencies [20,21]. Moreover, CBAs are time- and resource-intensive and thus only available in certain specialized laboratories. Alternative assays, including a novel immune-dot assay, have therefore been proposed; however, larger cohorts and multicenter studies are needed to validate these methods [24]. 

## 4. Neurofascin 155

NF antibodies were originally identified in immune-mediated axonal injury of patients with multiple sclerosis [25] and, shortly thereafter, described in a small cohort of patients with CIDP [26]. However, since then, a growing body of evidence has suggested a distinct clinical phenotype and disease course in patients harboring these antibodies. Antibodies recognizing NF155, the glial isoform of NF, are associated with a subacute to chronic onset and progressive symptoms reminiscent of distal CIDP with symmetric, predominantly distal weakness, sensory loss, and gait ataxia as well as tremor are characteristic for anti-NF155-mediated AN [19,27,28,29]. Yet, in contrast to CIDP, patients affected are predominantly males in young adulthood, with an increasing number of reports of pediatric cases resembling the adult clinical phenotype (see Table 1). The youngest case currently reported in detail encompasses the history of a 2-year and 3-month-old female patient presenting with progressive lower limb weakness for a period of 6 weeks and initially diagnosed with GBS [30].

Involvement of cranial nerves has been increasingly described, and further symptoms including autonomic dysfunction, neuropathic pain at onset, as well as papilledema are found in some individuals [19,36,39,40,41]. Furthermore, dysarthria, dysphagia, and cerebellar ataxia, symptoms reminiscent of brainstem encephalitis, have also been reported in patients with NF155-antibodies, overall hinting at potential CNS involvement [23,39].

In this context, Japanese studies have reported NF155 antibodies in patients exhibiting combined central and peripheral demyelination [23], although attempts to replicate these findings in Caucasian populations have yielded inconsistent results [42]. Moreover, Devaux and colleagues identified central demyelination in a subset (8%) of anti-NF155-IgG4-seropositive patients originally diagnosed with CIDP [43]. However, no clear link between antibody isotype and CNS involvement was found. Other studies that had detected anti-NF155-antibodies in the CSF, albeit at lower titers than in serum, in combination with other laboratory parameters have suggested blood–brain barrier disruption as mechanism for the presence of the antibodies in the CSF [19]. Notwithstanding, the existence of anti-NF155 antibodies in the CSF could provide a potential explanation for cerebellar symptoms in NF155-AN, although further studies examining CSF are needed to support this hypothesis [19]. 

### 4.1. Pathophysiology

Immunoglobulins targeting the glial NF isoform 155 located on myelin loops of Schwann cells and oligodendrocytes make up a large fraction of antibodies mediating AN [33,39]. Although the neuronal isoform 186 represents a co-target in a fraction of cases (see pan-NF), a notable number of patients harbor antibodies specific for NF155, thereby indicating the extracellular fibronectin type III (FnIII) domain 3, unique to NF155, as epitope in this AN [44,45].

The predominant immunoglobulin subclass is IgG4 [27,46]. Because of its structural inability to bind to C1q, the initial step in complement activation, and its low affinity for Fcγ receptors, it is assumed that IgG4-induced-NF155-AN is likely caused by the destruction of the paranodal complex [46,47]. In line with this, Koike et al. observed the absence of complement deposits at the paranodes, where deposits of NF155 IgG4 were identified [48]. Moreover, unlike classical, functionally monovalent IgG4 that recognize antigen with one Fab arm alone, a study conducted by Jentzer et al. demonstrated bivalency of anti-NF155 IgG4 as a pathogenic trait in animal models [49]. 

Further IgG subclasses, as well as IgM, have also been reported in a subset of patients [26,28,30,50]. Moreover, combinations of IgG4 and other immunoglobulins are frequently observed, and single cases of patients harboring IgG1, IgG2, and IgG3 titers alone have also been observed with slightly deviating disease courses and therapy responses in some studies [28,30,36,39]. In contrast, Delmont et al. found no significant correlation between demographic, clinical, or treatment data and antibody isotypes, with the exception of an older age at onset in patients with IgG4 in combination with other IgG isotypes versus IgG4 alone in younger patients (44a (42–47) versus 60a (54–67), *p* = 0.006) [50]. Differences in CSF protein concentration and outcome between IgG4 and IgG4-negative/IgM-NF155 patients have also been reported [41]. Yet, to date, the underlying mechanism and clinical significance of this observation remain uncertain. Although a potential immunoglobulin class and subclass switch has been proposed, in a longitudinal assessment of AN (up to >20a) serum, IgG switch was not observed [39]. Interestingly, a number of pediatric cases harboring IgG2 have been reported, albeit the exact relevance is currently unclear [30,36]. In general, further studies are needed to elucidate phenotypic correlations of immunoglobulin class and subclass, potentially shedding light on pathophysiology, and thereby providing treatment guidance and prognostic value.

### 4.2. Etiology

Although specific etiological factors of NF155-AN remain to be elucidated, an association between specific human leukocyte antigen (HLA) allele DRB1*15 (15:01 or 15:02) and patients with NF155-IgG4-AN compared with CIDP and controls has recently been identified, hinting at an HLA class II-restricted T-cell involved pathomechanism [19,51,52]. Higher incidences in East Asian [38,46] versus Western cohorts [50] point to this HLA association [51]. Moreover, only subtle clinical differences between HLA-DRB1*15:01 and HLA-DRB1*15:02 carriers have been identified, including a tendency of younger age in patients with DRB1*15:02 (45.2 ± 20.5 vs. 30.6 ± 14.6, *p* = 0.14) and more frequent CNS involvement in HLA-DRB1*15:02-DQB1*06:01 compared with HLA-DRB1*15:01-DQB1*06:02 carriers [19,51].

Prior infections have been reported in single patients, posing the question of a molecular mimicry-induced mechanism similar to that of GBS [30,33,50]. 

### 4.3. Pathology

Ultrastructural studies on NF155-IgG4-AN have so far identified significant widening of the nodes and periaxonal spaces, along with loss of transverse bands, thereby resulting in detachment of myelin loops and, ultimately, in disruption of the paranodal barrier [48,53]. Multifocal fiber loss, characterized by diminished large, myelinated nerve fibers, and varying overall reduction in myelinated fibers as well as subperineurial edema were also found in a subset of patients [41,46,48,53,54]. Onion bulbs are generally not observed. One study showed increased rates of segmental demyelination in biopsies of patients with NF155-IgG4, most often found in fascicular sciatic biopsy [41], while another group observed paranodal demyelination in <10% of teased myelinated nerve fibers in 2 biopsied patients [46]. While macrophage or inflammatory cells are usually scarce, highlighting a macrophage-independently mediated mechanism contrary to CIDP [48,54], Shelly et al. describe single endoneurial and epineurial inflammatory infiltrates identifiable in 81% (9/11), and one more severely affected patient showing a more widespread infiltration of epineurial mononuclear cells proximally than distally [41]. Myelin ovoids, providing evidence for axonal destruction, have also been identified, along with myelin thickening and variable degrees of paranodal separation in teased fiber preparations [41,48].

### 4.4. Paraclinical and Laboratory Findings

Paraclinical tests and laboratory parameters usually precede antibody testing and may aid the diagnosis of AN [6]. Electrophysiological results of NF155-AN show features consistent with demyelinating changes, commonly showing slowing of motor conduction velocities, prolonged distal motor latency, prolonged or absent F-waves, temporal dispersion, prolonged distal compound action muscle potential (CMAP) duration, and conduction block, with most patients meeting the EAS/PNS demyelinating electrophysiologic diagnostic criteria for CIDP [41,46,55].

Magnetic resonance imaging findings often show marked symmetric hypertrophy of cervical and lumbosacral roots/plexuses, with root diameters hinting at a positive correlation of disease duration, but this may be unremarkable [27,38,46]. A similar trend was observed in cranial nerve involvement, specifically the trigeminal nerves, although the clinically suspected involvement of optic nerves was not mirrored on MRI in one study investigating cranial nerve involvement [35].

CSF findings may also deviate from those found in CIDP. Patients with NF155-AN show significantly elevated CSF protein levels compared with seronegative CIDP patients [27,29,38]. In addition, Ogata et al. recently identified a distinct intrathecal chemokine profile in NF155-positive AN with a CIDP-like phenotype compared with classical CIDP, i.e., interleukin (IL)-13, CC motif chemokine ligand/eotaxin, and CXC motif chemokine ligand 8/IL8, with marked elevations of Th2-type cytokines [56]. The authors suggested that this increase in proinflammatory cytokines in the CSF may lead to disruption of the blood–nerve barrier at nerve roots, resulting in nerve edema and hypertrophy observed upon histology and MRI findings, respectively [35,56]. Furthermore, as mentioned above, some studies have found positive antibodies in CSF, with the significance of this observation not yet clear [19]. However, antibody titers are lower than in the periphery, and combined with the absence of oligoclonal bands, intrathecal antibody synthesis seems unlikely [19]. Moreover, lymphocytic pleocytosis is rare and, in most cases, instead, normal to discretely elevated cell count is mostly observed [19,27].

Discrepant data exist on the clinical correlation of antibody titers, with a recent study including a large cohort of NF155 AN showing a correlation of anti-NF155 antibody titers with modified Rankin scale results (r = 0.41, *p* = 0.004) and serum neurofilament when follow-up anti-NF155 antibody titers were evaluated using baseline titers as a reference, thereby illustrating potential biomarkers for monitoring disease activity [19,49].

### 4.5. Therapy

In contrast to CIDP, the response to IVIg in NF155 IgG4-mediated AN is usually poor, while treatment with B-cell depleting rituximab has a higher response rate in many patients, a phenomenon not uncommon in IgG4-mediated diseases [19,38,57]. In addition, a growing number of studies and case reports describe relapses after the initial response to therapy [27,39,57,58,59]. In this context, antibody subclass characterization may also play an important role in therapy guidance, as IgG3 > IgG1 > IgG2 antibodies activate complement and have been identified in a fraction of NF155-AN that might provide a rationale for a complement-targeting therapy in individual cases [21].

## 5. Neurofascin 186

The first observations of NF186 antibodies were reported by Devaux and colleagues in 2012 in patients diagnosed with GBS and CIDP, although not screened for cooccurrence with NF155 [5]. Since then, single descriptions of the presence of NF186 antibodies alone in patients with different clinical phenotypes have been reported [17,30,44], with larger cohorts only recently described, mainly in Chinese cohorts [32,60,61]. Age of onset is usually older than in NF155-AN, and, so far, cases of patients under the age of 20 are rare [30,32,61]. Two such cases entail 2-year-old male patients presenting with acute to subacute ascending weakness of the lower limbs and evidence of bulbar palsy in one (see Table 2).

Similar to NF155-AN, reports on clinical data of NF186-AN describe sensory motor polyneuropathy similar to GBS and CIDP. However, it must be noted that in earlier studies describing the presence of NF186, epitope distinction between NF186 and NF155 was not performed or was not explicitly described, rendering cross-study comparisons challenging [5,17,30].

Unlike NF155 AN, however, tremor is usually not observed, and onset is more often acute to subacute [44,61]. Although an NF186-AN-specific phenotype has not yet been elucidated, as with pan-AN, rare cases of glomerulosclerosis have been identified [44,63]. Furthermore, in a larger cohort, CNS involvement was described in approximately half of the patients, characterized by dizziness, impairment of vision, cranial nerve involvement and headache, as reported by Xie et al. [60], while in another study, signs of central demyelination on imaging were only present in one patient with concomitant CV2 antibodies [64].

### 5.1. Pathophysiology

Antibodies targeting the transmembrane protein NF186 bind to the extracellular Ig domains common to all NF as well as the isoform NF140 predominantly expressed in early development [32,44,65]. Older animal studies have indicated direct antibody pathogeneity in experimental autoimmune encephalitis models primed with human NF186, showing exacerbation of axonal injury and disease severity. Moreover, in NF186-knock-out mice, nodal disorganization was observed with loss of nodal Nav channels and AnkG enrichment, resulting in significantly reduced nerve conduction velocity [12]. In another animal study using spatiotemporal ablation, post-natal loss of NF186 resulted in gradual, progressive nodal destabilization and axonal degeneration in transgenic mice, alluding to a temporal component [66]. Indeed, ultrastructural observations of a patient with a CIDP-like phenotype also showed severe disorganization at an advanced stage, yet the exact mechanism and relevance are currently unknown [61].

Although IgG4 has been identified as a predominant subclass in many studies, findings from more recent cases show otherwise [30,36,61]. While Burnor et al. identified the presence of NF186-IgM in five patients with a CIDP-like phenotype, with a single patient showing additional low titers of IgG4 antibodies [32], Xie et al. found all three antibody classes in the majority of the cases [60]. It must be noted, however, that antibody isotype characterization has only been performed in single studies, and findings vary substantially, possibly because of in-house laboratory parameters [30,36,61,67].

### 5.2. Pathology

To date, only single reports describing the histopathological changes in NF186 exist [61,68]. A biopsy of a patient with NF186 antibodies reported by Liu et al. revealed a severe reduction in large and small myelinated fiber density in the absence of onion bulb formation [61]. T-cell, B-cell, and macrophage infiltrates were not observed. On ultrastructural examination, the extension of paranode length and fusing of paranode loops was noted. Vallat et al. described ultrastructural changes in detail in a patient with NF186; however, reactivity to NF155 was also noted and is therefore discussed in further detail in the section below [68].

### 5.3. Paraclinical and Laboratory Findings

Electrophysiological studies of patients with NF186-AN have revealed signs of demyelination characterized by reduced conduction velocity and axonal loss, which may occur both coincidingly or independently [30,60,61]. Conduction blocks have also been reported [60]. F-wave and distal motor latency that are prolonged but shorter than in NF155-AN have been noted [61].

Abnormalities in brachial plexus MRI were rare in one study [61], whilst the pediatric patient described by Harris et al. revealed enhancement in the nerve roots of the cauda equina [30]. Increased T2-weighted signals in the cerebellum, cerebral white matter, and brain stem, as well as in the meninges and spinal cord, have been described in single cases with CNS involvement [60].

CSF testing has so far revealed elevated protein levels in a large proportion of patients (75%) [61] in addition to WBC count in patients with CNS involvement versus PNS manifestation alone in a study by Xie et al. [60].

### 5.4. Therapy

Varying response rates to IVIg treatment possibly attributable to the immunoglobulin subclass, in combination with corticosteroids, have so far been reported, although leading to improvement in some cases and, again, potentially mirroring the relevance of the antibody subclass [30,32,61,63]. The pediatric patient described by Harris et al. responded well to IVIg therapy but relapsed at 3 months, following subsequent treatment with corticosteroids with initial improvement, and experienced yet another relapse at around 8 months and only slow improvement thereafter [30]. Therapy with rituximab has so far shown a good response [61].

## 6. Pan-Neurofascin

Pan-NF-AN represents a rare type of AN presenting with the following distinct clinical phenotype: patients are predominantly male, >60a with an acute onset, rapidly ascending symmetric sensorimotor weakness, reminiscent of GBS, and not uncommonly resulting in tetraplegia [69,70]. In contrast to NF155-AN, often characterized by a milder disease phenotype and younger age of onset, tremors and ataxia manifest less frequently [70]. Furthermore, pan-NF has a higher morbidity and mortality rate than NF155-AN [67]. In addition, cranial nerve involvement and autonomic instability with respiratory failure, as well as neuropathic pain, are also often observed [70]. Moreover, nephrotic syndrome, as mentioned above, is thought to be caused by the expression of NF186 in the renal glomeruli and represents a frequently cooccurring symptom of pan-NF-AN [69,71]. Pediatric reports are rare and clinically grossly resemble the adult phenotype (see Table 3).

### 6.1. Pathophysiology

Pan-NF antibodies co-target the glial NF isotype NF155 and the neuronal isotype NF186, both expressed in adult nodes, with prevalent reactivity to NF186 [44]. Six immunoglobulin-like domains, common to all NF-isoforms, represent the main epitopes of pan-NF antibodies, thereby permitting direct access to the node of Ranvier, as well as the FnIII domains 3 and 4, thought to be unique to NF155 [32,44,67]. As briefly mentioned above, pan-NF antibodies were first described in a small cohort of patients with MS by Mathey et al. in 2007, and their direct pathogeneity, i.e., inducible axonal damage, impairment of neuronal conduction, and clinical exacerbation, thereby assumed [25]. Direct access to the node, likely due to the nodal NF186 epitope, has been demonstrated and thought to be of pathophysiological relevance [67]. Moreover, a recent animal study investigating the intraneural passive transfer of pan-NF-IgG3 demonstrated an increased nodal length at the nerve roots of rats [72]. 

As immunoglobulin subclasses are found in various constellations, it is yet unclear which subclass plays the predominant pathogenic role. Initially, IgG3 and IgG4 were thought to represent the predominant subclass as these immunoglobulins were described in different reports [44,70]. Since then, however, IgG1 in patients with a severe disease course has been reported [69], as well as the cooccurrence of IgG2 in various cohorts [32]. Furthermore, the presence of IgG1 and IgG3—immunoglobulin subclasses that activate complement more strongly than IgG2 and IgG4, which have only limited complement activation potential—has been reported to lead to a more severe course and a relatively better response to IVIg therapy [69,70]. Although complement binding assays using patients’ derived pan-NF IgG3 have revealed complement deposition, a complement-mediated aggravation of the disease, rather than a complement-dependent cause, has been proposed as in vitro preincubation experiments have demonstrated a directly pathogenic role of pan-NF-antibodies, in the absence of humoral complement factors, and data on subclass and course deviate among studies [35,62,68,70]. Therefore, it has also been argued that the major phenotype determinant is the epitope itself, rather than the IgG subclass [70]. Naturally, further studies looking at long-term profiles are needed for elucidation. 

Interestingly, a recent case study identified pan-NF antibodies predominantly of the IgG2 subclass in two five-year-old male patients presenting with GBS- and CIDP-like symptoms with partial response to IVIg and steroids and prolonged recovery of up to 4 years [30]. Class switching, as observed in CNTN1-AN, and general subclass differences in adults and children may offer an explanation [30,67]. Indeed, Stengel et al. observed transiently detectable minor titers of IgG4 during the peak of IgG3 throughout the disease course of a severely affected patient with tetraplegia and near to locked-in syndrome [70]. In contrast, in a longitudinal assessment, Broers et al. reported the presence of IgG1, 2, and 3, with predominance of the latter in a 3-year-old toddler with a severe and prolonged disease course without observing a subclass switch [39]. 

### 6.2. Pathology

Only a few histopathological and ultrastructural reports of patients with pan-NF antibodies exist. Varying degrees of axonal loss have been reported, in the absence of cellular infiltration, inflammation, and onion-bulb formations [68,69,73]. In contrast, a biopsy specimen of a patient with a fulminant disease course showed single inflammatory T-cell infiltrates and increased floridity [74]. While segmental demyelination and signs of paranodal retraction or detachment were absent in two patients reported with IgG1 pan-NF antibodies [69], discrete demyelinating changes and single clusters of regeneration in a patient with pan-NF antibodies predominantly of the IgG3 subclass have also been described [68,74]. Ultrastructural analysis of a patient presenting with the latter and categorized as NF186-positive, although NF155 antibodies were also identified, demonstrated a varying degree of loss of Schwann cell microvilli as well as damage to the node of Ranvier, providing indirect evidence of pathogenicity, contrary to the normally appearing paranodal regions [68].

### 6.3. Paraclinical and Laboratory Findings

Electrophysiological findings demonstrate slowing of conduction velocity and conduction block without temporal dispersion, suggestive of nodal pathology [69,73]. Additionally, nerve inexcitability was reported in one patient two weeks after onset [69]. Throughout the disease course, signs of severe axonal degeneration with unrecordable sensory nerve action potentials (SNAPs) and CMAP have been described [69,73,75]. Moreover, electromyography may show abnormal spontaneous activity with fibrillation and positive sharp waves [74].

So far, reports on imaging in patients with pan-NF AN are scarce, with current descriptions including no apparent alterations on MRI in approximately 50% of patients and plexus and/or root abnormalities as well as in the cauda equina in the other half [30,69,74]. 

CSF protein levels may vary, with reports of mild elevation at onset and more severe albuminocytologic dissociation in other cases, while cell count is within the normal range [30,69,73,74]. Antibody titers may be found in the CSF, though lower than in serum, and oligoclonal bands have not been observed, again, suggesting a blood–brain barrier leakage rather than intrathecal synthesis [70,73,74]. Further serological findings include elevated levels of serum neurofilaments, which are thought to correlate with severity and outcome intra-individually [67].

### 6.4. Therapy

The administration of IVIgs may at first lead to improvement in some patients, in both adults and children; however, fulminant relapse has been observed within a few days or weeks [30,67,68]. Moreover, Harris and colleagues reported gradual and slow recovery upon treatment with IVIg in two pediatric cases with IgG2 antibodies, with relapse of symptoms in therapy absence in one of the patients [30]. Similarly, response to plasmapheresis is poor, while patients treated with rituximab have shown significant improvement [67]. Moreover, Fels et al. report improvement in clinical symptoms in a patient with predominant IgG4 pan-NF antibodies upon therapy escalation with the proteasome inhibitor bortezomib, thereby aiming at the entire B-cell axis, as well as T-cells and mononuclear phagocytes [74].

## 7. Contactin-1

Antibodies targeting CNTN1 were first described in 2012 by Devaux et al. in sera of a small subset of patients presenting with GBS-like and CIDP-like symptoms using combined cell-binding and nerve-binding assays [5]. They were further validated by Querol in 2013 using immunoprecipitation and CBAs in two patients with an acute, aggressive symptom onset mimicking CIDP with predominant motor involvement and poor response to IVIg [3]. Since then, several studies have shown a similar clinical phenotype in patients with CNTN1-AN, often presenting with acute to subacute disease onset with distal motor weakness and sensory ataxia, which is more prominent in males than females [50]. Although less frequent than in NF155-AN, tremor is more commonly found than in CIDP, and cranial nerve involvement with facial paralysis has also been reported in a substantial number of patients hinting at potential CNS involvement [33,50]. Nephrotic syndrome with proteinuria and hypoalbuminemia, as well as changes in the glomerular filtration rate, has additionally been reported in approximately 50% of patients, with immune complex deposits in renal biopsies [55,76,77].

While the age of disease of onset is usually towards the fifth or sixth decade, single cases of affected children have been described, clinically resembling the adult phenotype with ataxia and neuropathic pain (see Table 4) [3,34,39,62].

### 7.1. Pathophysiology

The glycosylphosphatidylinositol-anchored protein CNTN1 shares properties with NF as both have six Ig domains and four Fn domains. It interacts with Caspr1 at the endoplasmatic reticulum, thereby forming a complex important for the function and stability of the paranode [13,79]. Moreover, trafficking of CNTN1 to the paranode is Caspr1-dependent [79,80].

Binding of antibodies to the Ig domain of CNTN1 is thought to induce damage to the paranodal architecture, with controversial evidence of N-glycans within the IgG5-6 domain representing the main epitope [81,82]. In myelinating cultures of dorsal root ganglia neurons, CNTN1 antibodies lead to disruption between CNTN1/Caspr1 and NF155, resulting in paranodal alterations in myelinated fibers [81]. These changes are thought to be complement-independent and happen in the absence of inflammatory cells [81]. Moreover, a rodent study using the intraneural injection of patient-derived CNTN1-IgG4 showed induced paranodal destruction with lengthening of the node, resulting in conduction failure [83]. Further evidence for a direct pathogenic role was provided by Grüner et al., who showed a decrease in surface expression of CNTN1 and Na_v_ current densities on neurons upon chronic exposure to patient sera [84]. These studies also support the notion of a further site of attack, i.e., the dorsal root ganglia, potentially providing an explanation for certain symptoms like sensory ataxia [82,84].

Similar to NF155-AN, IgG4 has been identified as a prevailing subclass, although further classes and subclasses with currently unknown detailed significance have also been reported [50,82]. Indeed, IgG3 may be particularly present at disease onset, with complement deposition identified in CNTN1-IgG3-positive patients, which were bound by IVIg [85]. Similarly, in the above-mentioned study by Grüner and colleagues, more pronounced effects were observed in IgG3-predominant sera than in IgG4 [84]. Moreover, Doppler et al. compared the effects of IgG3 derived serum from a patient tested during acute onset versus IgG4 from a patient obtained several years into the disease in a rodent model, thereby concluding that acute disease onset may be IgG3-mediated, while IgG4 antibodies may lead to a more chronic course [86]. Further evidence for a pathological role of these antibodies is demonstrated upon histopathological examinations (see pathology). 

Similar to NF186-AN, associated nephropathy is attributed to the presence of CNTN1 expressed by podocytes of the kidney [87]. IgG4 deposits have been identified along the glomerular basement membrane in kidney biopsies of patients with CNTN1-AN [87]. In these patients, similar to the underlying neuropathy, the glomerulopathy responds poorly to IVIgs, while therapy response to rituximab is usually good.

### 7.2. Pathology

To date, histopathological findings of affected nerves in CNTN1-AN have revealed axonal loss and fiber degeneration in the absence of onion bulbs, as well as scant thinly myelinated fibers and prominent sub-perineurial edema, in contrast to biopsies of patients with CIDP [48,88,89]. Endoneurial presence of macrophages without T-cell infiltration was identified in some cases [88], whereas another study found no evidence for macrophage-induced demyelination, characteristic of CIDP [48]. Further ultrastructural findings include paranodal axo-glial detachment and widening of periaxonal space and, thereby, the impairment of saltatory conduction [48]. Moreover, skin biopsies examining myelinated fibers have shown elongated nodes and altered immunoreactivity signaling loss or destruction of paranodal Caspr1 and/or NF, which were less present in GBS or CIDP [88].

### 7.3. Paraclinical and Laboratory Findings 

Patients with anti-CNTN1 antibodies display severely abnormal nerve conduction, with slowed conduction velocities and prolonged distal latencies, consistent with signs of demyelination and often more pronounced than in CIDP, as well as early axonal involvement with varying reports of conduction block, similar to NF155-AN [55,62,83,89]. F-wave latencies are prolonged or absent, and CMAP duration is increased with a decreased amplitude [55].

Imaging data in these patients is scarce, with only few reports identifying thickening and gadolinium enhancement in nerve roots on MRI in most patients [90,91]. CSF protein levels are often highly elevated, whilst the CSF white blood cell count is mostly unremarkable [62,88,89].

### 7.4. Therapy

As initially observed, the response to IVIg is generally poor and is attributed to the predominant IgG4 isotype [82]. In this regard, IgG3 is strongly complement-inducing, and patients respond better to IVIg therapy. Indeed, Doppler et al. reported four patients with an acute onset of motor > sensory neuropathy relapsing to severe sensorimotor neuropathy, although initially responding well to IVIg therapy, of which two were screened in the acute phase and harbored predominant IgG3 [88]. In contrast, corticosteroids usually lead to a partial to good response, though with an unclear role [82,90]. In treatment-resistant patients, rituximab showed good and long-lasting response, which was also observed in children [34,39,57,88].

## 8. Contactin-Associated Protein 1

Antibodies binding Caspr1 were initially described in 2016 by Doppler and colleagues in two patients presenting severe neuropathic pain and a subacute onset, with a motor-dominant neuropathy resembling CIDP in one patient, while the other presented with a GBS-like phenotype [4]. Since then, only a few patients with Caspr1 have been reported, making this a relatively rare type of AN that accounts for 0.2–4% of CIDP diagnoses [4,44,50,59]. Symptoms are characterized by sensory ataxia, tremors, and a tendency to a subacute, rapidly progressive, and severe onset [33,39,50]. Neuropathic pain may be common but has not been reported in all cohorts, and cranial involvement similarly varies [33,39,50]. Furthermore, autonomic instability with respiratory failure has been reported in some cases [50].

Reports on age of onset vary, with some authors reporting younger onsets around the third decade [4,33,91], while others have identified a mean age of onset around the fifth decade [39]. Very few reports of cases under the age of 18 exist in the literature, two of which were mentioned by Cortese et al.: their study comprises the clinical characteristics of a 7-year-old female patient with a subacute onset and a 10-year-old female patient with a more chronic onset, both presenting with weakness of the lower limbs, and one with additional tremor and sensory ataxia (see Table 4) [33].

### 8.1. Pathophysiology

At the paranode, stabilized by the adapter protein 4.1B, the transmembrane glycoprotein Caspr1 forms a cis-complex with CNTN1 and is responsible for hindering the passage of nodal currents [13,92,93]. The exact antibody epitope is currently unknown; however, using purified antibodies from a Caspr1-seropositive patient in a cell aggregation assay and murine sciatic nerve fibers, Cortese et al. demonstrated an isotype-dependent penetration of the paranodal regions and binding of IgG4, but not IgG1, to the CNTN1/Caspr1 and NF155 complex, with assumed subsequent function-blocking activity and disruption of the axo–glial complex [33]. Indeed, both teased fibers and dermal fibers have shown lengthening of the nodal gab and disrupted paranodal regions [4]. Moreover, Doppler et al. showed a dispersion of sodium channels in the elongated nodes and paranodes, implying a morphological correlate of impaired nerve conduction, with the exact mechanism thus far unknown [4].

Similar to NF155- and CNTN1-AN, IgG4 has so far been identified as a prevailing subtype [33,39], which, however, has been associated with the more chronic form of the disease, while IgG3, also found in many patients, is thought to be found in more acute to subacute stages [4,94]. In line with this, complement deposition was observed in complement-binding assays of an IgG3-positive patient, while no such depositions were observed in a patient with IgG4 [4]. 

Neuropathic pain may be caused by affection of dorsal root ganglia, as initially proposed by Doppler et al. [4]. Analgesic therapy escalation with pregabalin and opioids was needed in the two initially described patients, whose sera reacted with small transient receptor potential cation channel subfamily V member 1-positive neurons in cultures of dorsal root ganglia, though to be involved in pain perception [4].

### 8.2. Pathology

Subperineurial edema along with severe axonal loss and degeneration have been described in sural nerve biopsies [4]. Features characteristic of de- or remyelination, i.e., onion bulbs and thinly myelinated fibers, were not observed in these patients, and T-cell infiltration was scarce.

### 8.3. Paraclinical and Laboratory Findings

Electrophysiological examinations in patients with Caspr1 show slightly varying results, with normal to slowed nerve conduction velocity, as well as prolonged distal motor and F-wave latency [4,22]. However, compound muscle and sensory nerve action potentials were shown to be preserved, all in all, indicating a demyelinating neuropathy [4,22]. Additionally, conduction block has also been reported [22,36]. 

CSF analysis reveals highly elevated protein levels [4,22,36]. Moreover, symmetric root hypertrophy and marked T2-signal hyperintensity have been reported on MRI [4,91].

### 8.4. Therapy

As seen in other ANs, the administration of IVIg shows a low response rate in most patients with few exceptions, potentially mirroring antibody isotypes [4,33,39,50,94]. Steroids has proven beneficial when administered in combination with further immunotherapy, and the response to rituximab is generally good [4,33,94].

## 9. Contactin 1/Contactin-Associated Protein 1 Complex

It remains uncertain whether or not antibodies targeting solely the CNTN1/Caspr1 complex constitute a separate AN subgroup compared to isolated Caspr1-AN or CNTN1-AN. Querol et al. initially described a patient presenting with sensory disturbances and weakness, showing electrophysiological features fulfilling the criteria of CIDP [3]. On examination of the patient’s serum using CBA, antibodies bound solely to cells co-expressing the CNTN1/Caspr1 complex, but not when the two CAMs were transfected separately, leading the authors to postulate the presence of a conformational epitope of the complex [3].

Subsequently, Pascual-Goñi et al. aimed to provide further elucidation in the debate on epitope-specificity and, using CBAs, identified 15 patients with a CIDP-like clinical picture with immunoreactivity against the CNTN1/Caspr1 complex, but not to cells transfected with CNTN1 or Caspr1 alone [95]. In contrast, reactivity for Caspr1 and CNTN1/Caspr1 were each measurable using ELISA, leading the authors to conclude that the antibodies in fact primarily target Caspr1, yet with enhanced specificity upon co-transfection of both CAMs. Indeed, clinical, paraclinical, and other laboratory findings resemble those of Caspr1-AN, with half of the patients initially diagnosed as GBS, with ataxia, cranial nerve involvement, and neuropathic pain. Neurophysiological testing revealed features attributed to acquired demyelination with non-uniform slowing of motor nerve conduction and an increase in F-wave latency. Most patients also showed signs of acute denervation with spontaneous activity and low-amplitude CMAP, hinting at axonal involvement. The predominant IgG isotype identified in sera was IgG4 (10/13), while IgG3 was found in the rest. Elevation of CSF protein levels was identified in all patients, and nerve root enhancement was a common finding on MRI. Histopathological examination revealed subtotal loss of myelinated fibers, subperineurial edema, and a relatively unremarkable density of small axons. Inflammatory infiltrates were rare, and there were no onion bulb formations. The response to IVIg was usually poor, although three IgG3-positive patients showed a partial response. The majority (90%) patients treated with rituximab improved, albeit slowly and gradually.

Several aspects of this study can be seen as beneficial, as these findings raise questions about the sensitivity and specificity of diagnostic methods. In addition, they emphasize the use of a second diagnostic test, as recommended by the EAN/PNS, and urge diagnostic staff to be aware of variations.

## 10. Discussion and Conclusion

**Figure 1:**
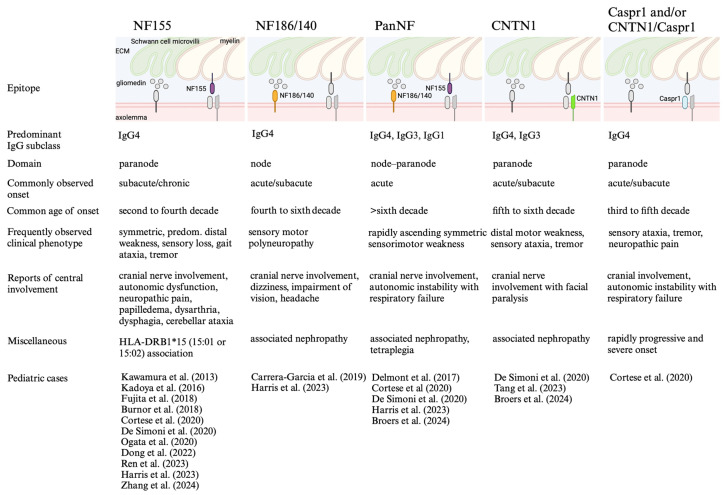
Summary of antigens, epitopes, clinical and paraclinical findings in autoimmune nodopathies, including list of pediatric cases [23,29,30,31,32,33,34,35,36,37,38,39,44,59,62,78]. Created in BioRender. Höftberger, R. (2024) BioRender.com/l96s138.

AN comprises a group of neuropathies that clinically resemble CIDP and GBS, with increasing evidence suggesting a different disease course and therapy response. Moreover, the age of disease onset seems to vary depending on the targeted epitope, with a trend towards earlier ages in certain AN subtypes, i.e., NF155-AN [19]. Isolated pediatric cases have also been described in other AN subtypes and generally resemble the adult phenotype [30,33,34,44]. This review uniquely summarizes previously described pediatric cases, an area that has not been explicitly explored so far. This is particularly relevant for pediatricians since the number of reports in children is increasing and the diagnosis has important implications for treatment and outcome. Moreover, we summarize current concepts along with experimental and clinical findings on ANs, offering clinicians and researchers an up-to-date overview of this topic (summarized in Figure 1). 

Particular attention should be paid to the diagnostic methods that are used for the detection of nodal/paranodal antibodies. Unless otherwise stated, this review focused on studies where patient reports were based on CBA, ELISA, and flow cytometry results, as recommended by the EAN/PNS (2022), thereby assuming high specificity and sensitivity in the diagnosis of the respective AN. Results using alternative methods, e.g., Western blot alone, were regarded as less reliable because of the loss of epitope confirmation and were therefore not included. Since CBAs are time- and resource-intensive, their implementation in a diagnostic laboratory can be challenging. Therefore, diagnosis may only be accessible in specialized laboratories, which might mask a substantially higher number of AN cases in both children and adults.

Another aspect that might contribute to differing frequencies lies in the patient cohorts themselves. While some studies recruited the sera of patients fulfilling diagnostic criteria of different neurological societies [4,36,39], others report findings from a broader range of patients [60,89]. Furthermore, previous studies were often conducted on pre-existing highly selected sera instead of samples collected in a manner reflecting clinical practice [50]. This approach adds to the challenge of comparing cohorts, and it was not fully considered when scanning the literature, potentially containing a bias regarding frequencies. Additionally, geographical and/or ethnic differences may play a role in varying incidences [50]. This latter aspect may be reflected in, and add valuable information to, the currently underexplored etiology of these disorders. In this regard, a certain HLA association with NF155-AN has been identified [51,52], with substantially higher frequencies found in East Asian versus Western populations [38,46,50]. Other etiological factors such as infections, as seen in classical GBS, have only rarely been identified in AN [5,34,37] Some of the ANs were reported to show a combined central and peripheral nervous system involvement, and patients may present an elevated CSF cell count and CSF protein as well as enhancement in nerve roots on MRI, supporting the hypothesis of intrathecal inflammation [23,56,96]. Whether this reflects a specific phenotype of patients with nodal/paranodal antibodies or more of a coincidence is currently unclear and requires further investigation. In this regard, the breakdown of the blood–brain and blood–nerve barriers, with potential exposure to certain antigens and subsequent antibody synthesis, could be taken into consideration in patients with concomitant inflammatory diseases.

Another important field in AN is the characterization of specific antibody classes and subclasses, which may provide valuable information on disease activity and the treatment response [21]. For example, IgG4 is found in the majority of ANs, providing a potential explanation for the limited IVIg therapy response in many patients and better outcomes upon B-cell depletion with rituximab [27,29,46,73]. In contrast, antibodies of the subclass IgG3 were found in a subset of ANs during the initial stage [85]. Complement deposition is associated with the latter, and cases respond well to IVIg therapy [85]. The evolution of immunoglobulins due to an indirect class switch was hypothesized but could not be confirmed in longitudinal studies [39,94]. Moreover, certain reports include the presence of IgG2 antibodies to different NF isoforms in pediatric patients; however, the relevance of this phenomenon is still unclear [30].

Taken together, ANs comprise a novel group of antibody-mediated neuropathies characterized by a distinct clinical and paraclinical phenotype reminiscent of CIDP or GBS [3,4,5]. While often fulfilling the electrophysiological criteria of CIDP, ANs often present with different disease courses and therapy responses. Moreover, certain constellations of symptoms and paraclinical as well as laboratory findings may point at the specific epitope, thereby prompting clinicians to search for AN and guide testing and therapy decisions, respectively. Although these diseases are mainly described in adults, increasing numbers of pediatric cases have been reported in recent years [30,34]. Clinical and paraclinical descriptions resemble those of adult findings; however, further studies are needed to characterize these phenotypes in children. In contrast, laboratory parameters, above all, antibody status including isotype characterization, may aid individualized therapy guidance and contribute to the elucidation of differences in pathophysiology in children.

## Figures and Tables

**Table 1:** Description of clinical, paraclinical (electrophysiology and magnetic resonance imaging), and laboratory data in published studies on anti-neurofascin155-seropositive pediatric patients.

Reference	Age (Years)	Sex	Antibody Epitope	Subtype/ Subclass	Testing Method	Titer at Diagnosis	Onset	Duration (Months)	Initial Diagnosis	Clinical Features	Electrophysiology	MRI	CSF-Protein Levels (mg/dL)	Therapy (Response)	Record of Prior Infection
Kawamura et al. (2013) [23]	16	f	NF155	N/A	ELISA, CBA	N/A	acute to subacute	N/A	CCPD, CIDP	progressive weakness of lower limbs, sensory ataxia, hyporeflexia	prolonged distal latency, CV slowing, CB, prolonged F-wave latency	brain: multifocal WML, gad. enhanced; cauda equina: gad. enhanced, hypertrophy	205	corticosteroids (CNS +, PNS -), IVIg (+), PE (+), Azathioprine + low-dose corticosteroids (+, stable)	N/A
Kadoya et al.	15	m	NF155	IgG4	ELISA, CBA	1:800	N/A	N/A	DADS	muscle weakness in lower limbs	N/A	N/A	199	N/A	N/A
(2016)	16	m	NF155	IgG4		1:800	N/A	>50	CIDP	gait ataxia, tremor	N/A	N/A	366	IVIG (-)	N/A
[29]	18	m	NF155	IgG4		1:1600	N/A	14	DADS	dysesthesia in lower limbs	N/A	N/A	135	IVIG (-)	N/A
Fujita et al. (2018) [31]	16	m	NF155	N/A	Flow cytometry	N/A	subacute	>28	CIDP	distal predominant weakness of the lower limbs, sensory ataxia, postural tremor, hyporeflexia	prolonged distal and F-wave latencies, decreased CV	hypertrophy of the cervical and lumbosacral roots/plexus	412	IVIg (-), Methyl-prednisolone (+)	N/A
Burnor et al. (2018) [32]	13	f	NF155	IgG4 > IgG1 and IgG2; IgM	CBA	1:200	chronic	>6	CIDP	inability to walk, multifocal numbness, areflexia	N/A	N/A	N/A	IVIg and apheresis (?), Prednisone (+)	N/A
Cortese et al. (2020) [33]	13	f	NF155	IgG4	ELISA, CBA	N/A	subacute	N/A	CIDP	lower > upper limb weakness, sensory ataxia, tremor,	N/A	N/A	N/A	IVIg (+), steroid (+)	N/A
De Simoni et al. (2020) [34]	7.9 (3–11)	3:2 (m:f)	2 panNF, 1 NF155, 2 CNTN1	IgG4 predom., (100–75%)	Flow cytometry	N/A	chronic	N/A	CIDP	ataxia (5/5), neuropathic pain (4/5), tremor (3/5; 2 CNTN1+ and 1 panNF+) cranial nerve involvement and optical neuritis in 1 CNTN1+	N/A	N/A	292.4 (75–619)	IVIG (-/partial)	1/5 (gastro-intestinal infection)
Ogata et al. (2020) [35]	13	m	NF155	IgG4	Flow cytometry	N/A	N/A	N/A	DADS	N/A	N/A	hypertrophy of lumbo-sacral and cervical nerve roots	205	N/A	N/A
Dong et al. (2022) [36]	16	f	NF155	IgG4 > IgG2 and IgG1	CBA	N/A	chronic	21	CIDP	symmetric proximal and distal muscle weakness, hyporeflexia, tremor, sensory ataxia, autonomic symptoms, cranial nerve involvement	not specified for individual case	N/A	134.19	not specified for individual case	N/A
	10	m	NF155	IgG4 > IgG2		N/A	chronic	48	CIDP	symmetric proximal and distal muscle weakness, hyporeflexia, tremor, sensory ataxia		N/A	184.92		N/A
	15	m	NF155	IgG4 and IgG2		N/A	chronic	7	CIDP	symmetric proximal and distal muscle weakness, hyporeflexia, tremor, paresthesia, sensory ataxia		N/A	287.28		N/A
Ren et al. (2023) [37]	14	f	NF155	IgG4	CBA	initially 1:10, later 1:1000	subacute	>12	CIDP	progressive muscle weakness, distal numbness, dysphagia, hypoesthesia, hyporeflexia, tremor, ataxia	signs of demyelination and axonal damage	unremarkable	182	PE (relapse); RTX (modest +), AI+, corticosteroids (+ but side effects), Telitacicept (+)	cold
Harris et al. (2023) [30]	2	f	NF155	IgG4 and IgG1 > IgG2	CBA	1:800	subacute	>10	GBS	progressive lower limb weakness, shoulder girdle weakness	abnormal sensory and motor responses, absent and prolonged F-waves	1 year after onset mild cauda equina root enhancement	190	IVIg (-), Prednisolone (+)	N/A
Zhang et al. (2024) [38]	16	m	NF155	N/A	CBA	N/A	acute	5	CIDP	weakness of limbs, sensory dysfunction, ataxia, cranial neve involvement (facial)	signs of demyelination and axonal damage, CV reduction, prolonged DML, CMAP amplitude reduction in both patients	diffuse thickening of the lumbosacral nerve roots	352	IVIg (-), PE (+), RTX (+)	N/A
	15	m	NF155	N/A		N/A	chronic	3	CIDP	weakness of limbs, sensory dysfunction, ataxia,		N/A	337	PE (+)	N/A

Abbreviations: CB = conduction block; CBA = cell-based assay; CCPD = combined central and peripheral demyelination, CIDP = chronic inflammatory demyelinating polyneuropathy; CMAP = compound muscle action potential; CNTN1 = contactin1; CV = conduction velocity; DML = distal motor latency; DADS = distal acquired demyelinating symmetric neuropathy; ELISA = enzyme-linked immunosorbent assay; gad. = gadolinium; GBS = Guillain–Barré syndrome; IgG = Immunoglobulin G; IVIg = intravenous immunoglobulin; N/A = not available; NF155 = neurofascin 155; panNF = pan-neurofascin; PE = plasma exchange; RTX = rituximab; + = positive; (+) = good response; (-) = poor response.

**Table 2:** Description of clinical, paraclinical (electrophysiology and magnetic resonance imaging), and laboratory data in published studies on anti-neurofascin186/140-seropositive pediatric patients.

Reference	Age (Years)	Sex	Antibody Epitope	Subtype/ Subclass	Testing Method	Titer at Diagnosis	Onset	Duration (Months)	Initial Diagnosis	Clinical Features	Electrophysiology	MRI	CSF-Protein Levels (mg/dL)	Therapy (Response)	Record of Prior Infection
Carrera-Garcia et al. (2019) [62]	2	m	NF186/140	N/A	CBA	N/A	acute	1, relapse 1 year later	CIDP	weakness and areflexia of lower limbs	conduction slowing, CMAP amplitude decrease, prolonged distal latencies	N/A	125	IVIg (mild +, relapse), methyl-prednisolone (+)	diarrhea reported 3 days prior to symptom onset
Harris et al. (2023) [30]	2	m	NF186/140	IgG2	CBA	N/A	chronic	>4	N/A	progressive weakness of lower and upper limbs, evidence of bulbar palsy	abnormal sensory and motor responses, demyelinating changes	enhancement in the cauda equina nerve roots	105	IVIg (+)	N/A

Abbreviations: CBA = cell-based assay; CIDP = chronic inflammatory demyelinating polyneuropathy; CMAP = compound muscle action potential; IgG = Immunoglobulin G; IVIg = intravenous immunoglobulin; N/A = not available; NF186 = neurofascin 186/140; (+) = good response.

**Table 3:** Description of clinical, paraclinical (electrophysiology and magnetic resonance imaging), and laboratory data in published studies on anti-pan-neurofascin-seropositive pediatric patients.

Reference	Age (Years)	Sex	Antibody Epitope	Subtype/ Subclass	Testing Method	Titer at Diagnosis	Onset	Duration (Months)	Initial Diagnosis	Clinical Features	Electrophysiology	MRI	CSF-Protein Levels (mg/dL)	Therapy (Response)	Record of Prior Infection
Delmont et al. (2017) [44]	2	m	panNF	IgG4 > IgG2	ELISA, Western blot, CBA	NF186 1:1000, NF140 1:2000, NF155 1:2000	subacute	>5	CIDP	weakness of lower limbs, hyporeflexia	demyelinating changes, CV	N/A	N/A	IVIg (+), steroids (-)	0
Cortese et al. (2020) [33]	2	m	panNF	IgG3	ELISA, CBA	N/A	subacute	N/A	CIDP	lower limb weakness, gait instability	N/A	N/A	N/A	IVIg (+)	N/A
De Simoni et al. (2020) [34]	7.9 (3–11)	3:2 (m:f)	2 panNF, 1 NF155, 2 CNTN	IgG4 predom., (100–75%)	Flow cytometry	N/A	chronic	N/A	CIDP	ataxia (5/5), neuropathic pain (4/5), tremor (3/5; 2 CNTN1+, 1 panNF+) CN involvement and optical neuritis in 1 CNTN1+	N/A	N/A	292.4 (75–619)	IVIg (-/partial)	1/5 (gastro-intestinal infection)
Harris et al. (2023) [30]	5	m	panNF	IgG2	CBA	N/A	acute	>7	CIDP	progressive ataxia, weakness of lower limbs, areflexia	demyelinating changes	enhancement in bilateral CN III, cervical root and cauda equina nerve root	230 (initially); 470 (follow up 5 weeks)	IVIg (initial +, response reduced during relapses)	tick bite 10 days prior to symptom onset
	5	m	panNF	IgG2		N/A	subacute	>48	GBS (initally), CIDP (reclassified)	progressive lower limb weakness and areflexia	initially unremarkable except from absent F-wave responses	unremarkable	52	IVIg (+), RTX (+)	N/A
Broers et al. (2024) [39]	3	m	panNF	IgG3 > IgG1 and IgG2	CBA	IgG 1:1600	acute	N/A	GBS	severe muscle weakness, ataxia, pain	N/A	N/A	elevated	IVIg and methyl-prednisolone (-), RTX and steroids (initial + with relapse after 2 months); long-term RTX, PE and cyclophosphamid (+)	N/A

Abbreviations: CBA = cell-based assay; CIDP = chronic inflammatory demyelinating polyneuropathy; CN = cranial nerve; CNTN1 = contactin 1; CV = conduction velocity; ELISA = enzyme-linked immunosorbent assay; GBS = Guillai–Barré syndrome; IgG = Immunoglobulin G; IVIg = intravenous immunoglobulin; N/A = not available; NF155 = neurofascin 155; panNF = pan-neurofascin; PE = plasma exchange; RTX = rituximab; + = positive; (+) = good response; (-) = poor response.

**Table 4:** Description of clinical, paraclinical (electrophysiology and magnetic resonance imaging), and laboratory data in published studies on anti-contactin1-seropositive and anti-Caspr1-seropositive pediatric patients.

Reference	Age (Years)	Sex	Antibody Epitope	Subtype/ Subclass	Testing Method	Titer at Diagnosis	Onset	Duration (Months)	Initial Diagnosis	Clinical Features	Electrophysiology	MRI	CSF-Protein Levels (mg/dL)	Therapy (Response)	Record of Prior Infection
De Simoni et al. (2020) [34]	7.9 (3–11)	3:2 (m:f)	2 panNF, 1 NF155, 2 CNTN	IgG4 predom., (100–75%)	Flow-cytometry	N/A	chronic	N/A	CIDP	ataxia (5/5), neuropathic pain (4/5), tremor (3/5; 2 CNTN1+ and 1 panNF+) cranial nerve involvement and optical neuritis in 1 CNTN1+	N/A	N/A	292.4 (75–619)	IVIg (-/partial)	1/5 (gastro-intestinal infection)
Tang et al. (2023) [78]	14	m	CNTN1	IgG1 and IgG4	CBA	1:10	acute	N/A	CIDP and nephropathy	weakness and numbness of lower limbs, ataxia, pain, autonomic symptoms, nephropathy	relatively unremarkable sensory CV and amplitudes of SNAPs in both lower and upper limbs	unremarkable	284	Steroids (+, relapse)	1
Broers et al. (2024) [39]	5	f	CNTN1	IgG4 > IgG1 and IgG2	CBA	IgG 1:6400	acute	N/A	CIDP	weakness of lower extremities, pain, tremor	N/A	N/A	elevated	IVIgs (-), RTX and prednisolone (+)	N/A
Cortese et al. (2020) [59]	7	f	Caspr1	IgG1	CBA	N/A	subacute	N/A	CIDP	proximal and distal weakness of lower limbs, postural and intentional tremor, sensory ataxia	N/A	N/A	N/A	IVIg (+), steroids (-)	N/A
	10	f	Caspr1	not detectable	CBA	N/A	chronic	N/A	CIDP	lower limb weakness, gait disturbances	N/A	N/A	N/A	IVIg (+)	N/A

Abbreviations: Caspr1 = contactin-associated protein 1; CBA = cell-based assay; CIDP = chronic inflammatory demyelinating polyneuropathy; CNTN1 = contactin 1; CV = conduction velocity; IgG = Immunoglobulin G; IVIg = intravenous immunoglobulin; N/A = not available; NF155 = neurofascin 155; panNF = pan-neurofascin; RTX = rituximab; SNAP = sensory nerve action potential; + = positive; (+) = good response; (-) = poor response.
